# Screening of Genes Related to Fat Deposition of Pekin Ducks Based on Transcriptome Analysis

**DOI:** 10.3390/ani14020268

**Published:** 2024-01-15

**Authors:** Bozhi Shi, Ziyue Zhang, Xueze Lv, Keying An, Lei Li, Zhaofei Xia

**Affiliations:** 1 College of Veterinary Medicine, China Agricultural University, Beijing 100193, China; shibozhi1998@163.com (B.S.); zzyue0211@126.com (Z.Z.); ankeying126@126.com (K.A.); 2 Beijing General Station of Animal Husbandry, Beijing 100107, China; lvxueze0310@163.com; 3 College of Veterinary Medicine, Yunnan Agricultural University, Kunming 650500, China

**Keywords:** Pekin duck, subcutaneous fat, slaughter performance, RNA-seq, fat metabolism

## Abstract

**Simple Summary:**

Fat deposition ability is an important index with which to evaluate meat-producing ducks. Ducks with the same diet and environment can present with differences in fat deposition ability if they have diverse genetic backgrounds. Nankou 1 Pekin ducks and Jingdian Pekin ducks are common strains of Pekin duck, a famous, large-sized meat-producing duck breed with a fast growth rate, high feed conversion rate and short growth cycle. The aim of this study is to investigate the differences in slaughtering performance and transcriptome profiles of different strains of Pekin ducks, so as to explore the mechanisms regulating fat deposition in ducks. The results showed that Nankou 1 had greater abdominal fat and subcutaneous fat content than Jingdian ducks, and that the gene expression differences between these two strains of ducks may be the cause of the differences in fat deposition. This study lays a foundation for exploring the regulatory mechanism of fat deposition in ducks and provides a theoretical basis for breeding high-quality meat-producing ducks.

**Abstract:**

Subcutaneous fat deposition is an important index with which to evaluate meat-producing ducks, and affects their meat quality and feed conversion rate. Studying the differentially expressed genes in subcutaneous fat will help to comprehensively understand the potential mechanisms regulating fat deposition in ducks. In this study, 72 Nankou 1 Pekin Ducks and 72 Jingdian Pekin Ducks (half male and half female) at 42 days of age were selected for slaughter performance and transcriptome analysis. The results showed that the breast-muscle yield of Nankou 1 ducks was significantly higher than that of Jingdian ducks, but that the abdominal fat yield and subcutaneous fat yield were higher than that of Jingdian ducks. Thousands of DEGs, including many important genes involved in fat metabolism regulation, were detected by transcriptome. KEGG enrichment analysis showed that the DEGs were significantly enriched on pathways such as regulation of lipolysis in adipocytes, primary bile acid biosynthesis, and biosynthesis of unsaturated fatty acids. SCD, FGF7, LTBP1, PNPLA3, ADCY2, and ACOT8 were selected as candidate genes for regulating subcutaneous fat deposition. The results indicated that Nankou 1 had superior fat deposition ability compared to Jingdian ducks, and that the candidate genes regulated fat deposition by regulating fat synthesis and decomposition.

## 1. Introduction

Pekin duck is a famous, large-sized meat-producing duck breed with a fast growth rate, high feed conversion rate and short growth cycle, and occupies an important position in the poultry market [1]. Pekin duck is the main source of Pekin Roast Duck; Nankou 1 is the highest market share strain at present, while Jingdian Pekin duck is a newly bred filler-free strain developed in 2021. Fat deposition in animals includes subcutaneous fat, abdominal fat and intramuscular fat. After fat is synthesized in the liver, it is transported and stored in the adipose tissue [2]. Subcutaneous fat deposition is a common slaughter performance index and important economic trait of meat ducks. A low subcutaneous fat percentage will affect the quality and taste of duck meat, while a high sebum percentage will reduce the feed conversion rate and thus increase the breeding cost [3]. Existing studies have screened out the differentially expressed genes of subcutaneous fat in different breeds of pigs, cattle, and sheep, and have identified the regulatory pathway of fat deposition on this basis [4,5,6]. Some studies compared the gene expression of subcutaneous fat in Muscovy ducks at different developmental stages, and screened out the genes related to fatty acid biosynthesis and fatty acid oxidation [7]. However, the underlying genetic mechanisms regulating subcutaneous fat deposition in Pekin ducks remains unclear.

The phenotypic differences exhibited by animals with similar genetic backgrounds may be initially explained by changes in the transcriptome. We tried to explore and explain phenotypic differences at the genetic level. With the maturity of next-generation high-throughput DNA sequencing technology, the efficiency of analyzing gene expression and exploring differentially expressed genes (DEGs) has been greatly improved [8,9]. Transcriptome sequencing (RNA-seq) can investigate the function and regulatory mechanisms of genes at the global level and mine idiosyncratic function-related candidate genes. Some scholars completed the assembly of the whole duck genome sequence with Pekin duck as the sample [10], and since then the duck reference genome has been constantly perfected [11,12], which has improved the reliability and accuracy of duck RNA-seq analysis. RNA-seq has been widely used in the studies of pigs [13], cattle [14], sheep [15], chickens [16] and other animals, and has successfully identified many key genes that regulate fat metabolism. However, studies on the transcriptome analysis of duck adipose tissue are few.

In this study, we compared the slaughter performance of Nankou 1 Pekin Ducks and Jingdian Pekin Ducks and performed RNA-seq on their subcutaneous fat tissue to identify important genes regulating subcutaneous fat deposition in ducks. This study provides a basis for further exploration of the molecular regulatory mechanisms of ducks.

## 2. Materials and Methods

### 2.1. Experimental Animals

All experimental procedures involving the manipulation of ducks were conducted in accordance with the “Guidelines for Experimental Animals” of the Ministry of Science and Technology (Beijing, China). This study was reviewed and approved by the Laboratory Animal Welfare and Animal Experimental Ethical Review Committee of China Agricultural University (Beijing, China) under permit No. AW71303202-2-1.

Seventy-two Nankou 1 Pekin Ducks (36 males and 36 females) and seventy-two Jingdian Pekin Ducks (36 males and 36 females) at 1-day-old with similar body weights were provided by Beijing Golden Star Duck Co., Ltd. (Beijing, China). All ducks were randomly divided into A group (Nankou 1, female), B group (Nankou 1, male), C group (Jingdian, female), and D group (Jingdian, male) according to strain and sex. There were 4 replicates per treatment group and 9 ducks per replicate. The ducks were fed and managed under the same environmental conditions with free access to feed and water and 24 h light. The temperature and humidity of the duck house were kept constant, so that the temperature was stable at 35 °C for the first week, and then gradually dropped to 20 °C. The humidity was kept above 60%.

### 2.2. Sample Collection 

At 42 days of age, 2 ducks from each replicate were randomly selected, weighed, and slaughtered after 8 h of fasting. The eviscerated weight was measured as the carcass weight after removal of the esophagus, trachea, spleen, gastrointestinal tract, crop, gallbladder, pancreas, gonads, liver, heart, glandular stomach, gizzard, and abdominal fat. The eviscerated carcass, thigh muscle, breast muscle, abdominal fat and subcutaneous fat were weighed separately to calculate slaughter performance. Subcutaneous fat was collected from all adipose tissue under the skin from the neck to the knee joint. The abdominal fat included all the fat in the abdominal cavity. The detailed determination of carcass characteristics refers to the agricultural industry standard NY/T 823-2020 [17], as follows:

Eviscerated yield (%) = (eviscerated weight/fasted live weight) × 100%,(1)

Breast muscle yield (%) = (breast muscle weight/eviscerated weight) × 100%,(2)

Thigh muscle yield (%) = (thigh muscle weight/eviscerated weight) × 100%,(3)

Abdominal fat yield (%) = [abdominal fat weight/(abdominal fat weight + eviscerated weight)] × 100%,(4)

Subcutaneous fat yield (%) = [(abdominal fat weight + subcutaneous fat weight)/eviscerated weight] × 100%,(5)

Subcutaneous fat was collected and placed in a freeze-deposit tube, stored in liquid nitrogen for a short time, and then transferred to an ultra-low temperature freezer at −80 °C for subsequent analysis.

### 2.3. RNA Isolation, cDNA Library Construction and RNA-Seq

Total RNA was extracted from the tissue using TRIzol^®^ Reagent (Invitrogen, Carlsbad, CA, USA) according to the manufacturer’s instructions. Then, RNA quality was determined by 5300 Bioanalyzer (Agilent, Beijing, China) and quantified using the ND-2000 (NanoDrop Technologies, Waltham, MA, USA). At least 1 μg of each RNA sample (1.8 < OD260/OD280 < 2.2, RNA integrity number ≥ 6.5) was used to construct the sequencing library.

RNA purification, reverse transcription, library construction, and sequencing were performed at Shanghai Majorbio Bio-pharm Biotechnology Co., Ltd. (Shanghai, China) according to the manufacturer’s instructions (Illumina, San Diego, CA, USA). The RNA-seq transcriptome library was prepared following Illumina^®^ Stranded mRNA Prep, Ligation from Illumina (San Diego, CA, USA) using 1 μg of total RNA. The RNA-seq sequencing library was sequenced by the Illumina sequencing platform (Nova Seq 6000 Illumina). 

The fastq software (version 0.23.3) was used to control sequencing quality and obtain clean reads. The clean reads were then mapped to the duck reference genome (http://asia.ensembl.org/Anas_platyrhynchos_platyrhynchos/Info/Index accessed on 1 April 2019) by HISAT2 software (version 2.2.1). 

### 2.4. Differential Expression, and Functional Analysis

To identify differential expression genes (DEG) between two different samples, the expression level of each transcript was calculated according to the transcripts per million reads (TPM) method. Essentially, differential expression analysis was performed using the DESeq2. DEGs with |log2FC| ≥ 1 and FDR < 0.05 were considered to be significantly different expressed genes. In addition, functional-enrichment analysis including GO and KEGG were performed to identify which DEGs were significantly enriched in GO terms and for metabolic pathways. Diamond (version 2.1.6) was used to analyze the gene ontology (GO) functions. KEGG pathway analysis was carried out by KOBAS 3.0. It is generally considered that GO terms and KEGG pathways with adjusted *p* < 0.05 are significantly enriched.

### 2.5. Quantitative Real-Time PCR Validation

qRT-PCR was used to verify the levels of expressed genes. Six differentially expressed genes were randomly selected for validation (Table 1), with glyceraldehyde-3-phosphate dehydrogenase (GAPDH) as the internal reference gene. Total RNA extracted from the tissues was reverse-transcribed into cDNA using an Evo M-MLV RT Mix Kit with gDNA Clean for qPCR Ver.2 (Accurate Biotechnology Co., Ltd., Changsha, China). SYBR Green Premix Pro Taq HS qPCR Kit III (Accurate Biotechnology Co., Ltd., Changsha, China) and an ABI StepOnePlus Real-Time PCR System (ABI 7500, Applied Biosystems, Waltham, MA, USA) were used for RT-PCR, and each sample was assayed three times. 

### 2.6. Statistical Analysis

Data were analyzed using the SPSS statistical software (version 26.0) and GraphPad Prism (version 9.0). Two-way ANOVA analysis was used to analyze the differences of slaughtering performance between groups. Gene expression was calculated using the relative quantification (2 − ΔΔCT) method. Graphs were drawn with Adobe Illustrator (version 2020). Data were presented as Mean ± SEM, and *p*-values below 0.05 and 0.01 were considered statistically significant and highly significant, respectively.

## 3. Results

### 3.1. Body Weight and Slaughtering Performance

As shown in Table 2, strain and sex factors had no interaction effect on slaughter performance, and there was no difference in body weight, eviscerated yield, and thigh muscle yield of Pekin ducks with different strains and sexes (*p* > 0.05). Both strain and sex factors had significant effects on breast muscle development. Compared with Nankou 1 ducks, the breast muscle yield of Jingdian ducks was significantly increased (*p* < 0.01), while the breast muscle yield of male ducks was also significantly higher than that of female ducks (*p* < 0.05). Abdominal fat yield was significantly affected by sex (*p* < 0.01), and it was lower in the Jingdian male duck group than in the female duck group. However, the subcutaneous fat yield was significantly affected by strain factors (*p* < 0.05), resulting in a higher subcutaneous fat yield in Nankou 1 male ducks than in Jingdian male ducks.

### 3.2. Overview of Sequencing Data

A total of 16 libraries (i.e., 4 replicate libraries per group with 2 ducks per replicate) were subjected to transcriptome sequencing. As shown in Supplementary Table S1, a total of 110.23 Gb of clean reads was obtained, and the clean reads of each sample were greater than 6.28 Gb. The Q30 value was higher than 93% for each sample, indicating that the sequencing quality was good and could be used for subsequent analysis. The percentages mapped to the reference genome were higher than 80% in all samples. Meanwhile, approximately 81.7% of the reads could be uniquely mapped to the Anas platyrhynchos genome (Supplementary Table S2).

### 3.3. Screening DEGs

**Figure 1:**
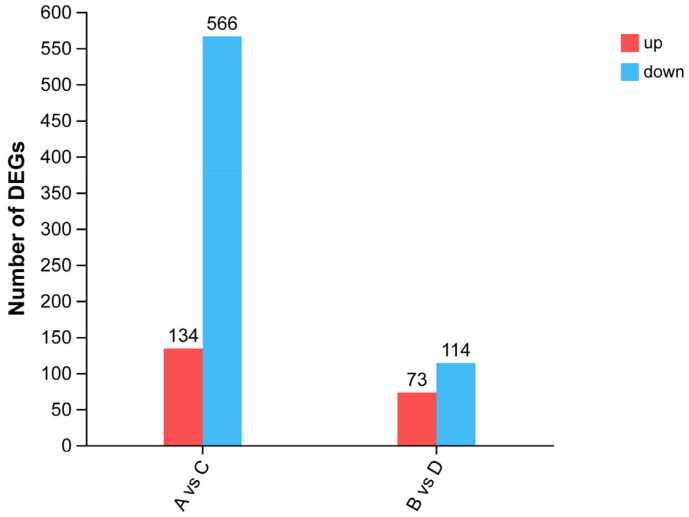
The number of DEGs.

According to the results of slaughter performance (Table 1), there were significant differences in subcutaneous fat deposition among the different strains of Pekin ducks. Therefore, the gene expression of Pekin ducks of different strains of the same sex was subsequently analyzed in this study. A total of 700 DEGs were screened and compared between groups A and C (Supplementary Table S3), among which 134 genes were upregulated and 566 genes were downregulated. Comparing group B and D, 187 DEGs were screened (Supplementary Table S4), including 73 upregulated genes and 144 downregulated genes (Figure 1).

**Figure 2:**
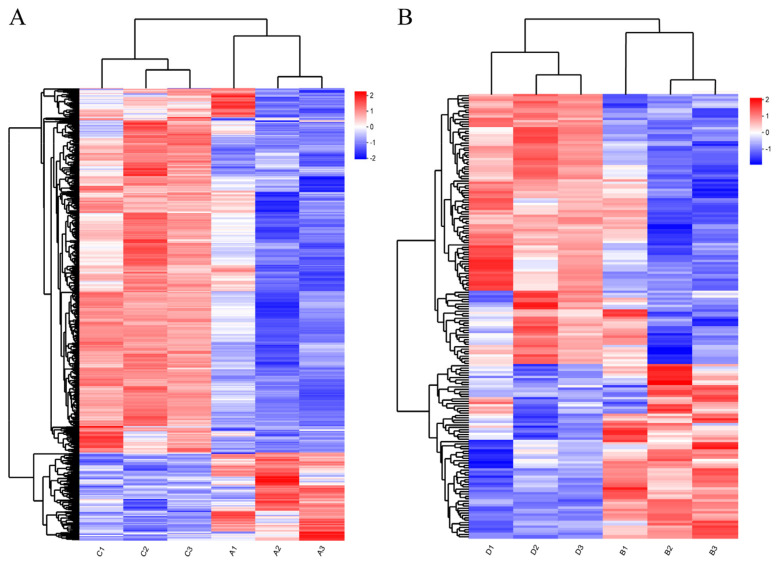
Hierarchical clustering analysis of DEGs: (**A**) hierarchical clustering analysis of DEGs between Nankou 1 female ducks (A group) and Jingdian female ducks (C group); and (**B**) hier-archical clustering analysis of DEGs between Nankou 1 male ducks (B group) and Jingdian male ducks (D group). The color in the heat map represents gene expression changes. Red indicates up-regulation of gene expression; blue indicates downregulation of expression.

The gene hierarchical clustering analysis of DEG expression patterns (Figure 2) indicated that the samples in each group had good repeatability and showed the reliability of the gene sets.

### 3.4. Functional Enrichment Analysis of DEGs

**Figure 3:**
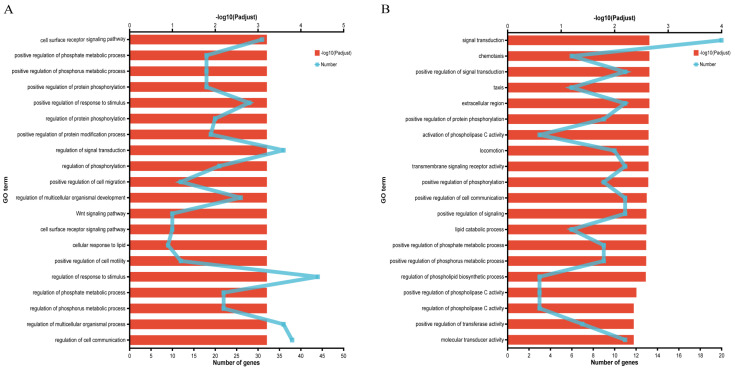
GO enrichment analysis of DEGs related to fat metabolism: (**A**) GO enrichment analysis of DEGs between Nankou 1 female ducks (A group) and Jingdian female ducks (C group); and (**B**) GO enrichment analysis of DEGs between Nankou 1 male ducks (B group) and Jingdian male ducks (D group).

GO enrichment analysis of DEGs from A vs. C group and B vs. D group screened 129 and 37 genes associated with fat metabolism, respectively (Supplementary Tables S5 and S6). Functional enrichment analysis showed that the DEGs from A vs. C group were significantly enriched in 228 GO terms (*p* < 0.05), including 190 GO terms in the biological process (BP) category, 10 GO terms in the cellular component (CC) category, and 28 GO terms in the molecular functions (MF) category, which were mainly enriched in cell surface receptor signaling pathway, regulation of phosphorylation, and cellular response to lipids, etc. (Figure 3A). At the same time, the DEGs from B vs. D group were significantly enriched in 72 GO terms (*p* < 0.05), including 59 GO terms in the BP category, 3 GO terms in the CC category, and 10 GO terms in the MF category, which were mainly enriched in signal transduction, activation of phospholipase C activity, lipid catabolic process, etc. (Figure 3B).

### 3.5. KEGG Pathway Analysis of DEGs

**Figure 4:**
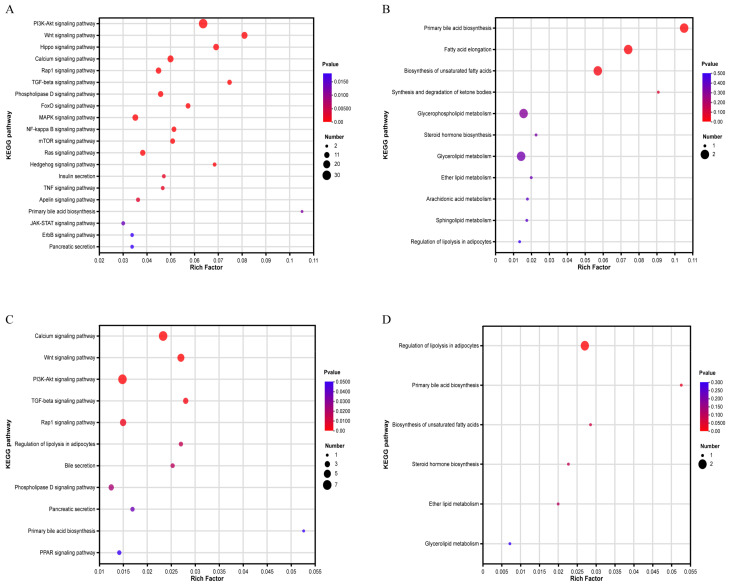
KEGG enrichment analysis of DEGs between Nankou 1 and Jingdian Pekin ducks: (**A**) significantly enriched pathway of DEGs between A and C groups; (**B**) fat-metabolism-related pathways of DEGs between A and C group; (**C**) significantly enriched pathway of DEGs between B and D groups; and (**D**) fat-metabolism-related pathways of DEGs between B and D groups.

KEGG enrichment analysis of DEGs from the A vs. C group revealed that genes were significantly enriched (*p* < 0.05) in signaling pathways such as the PI3K-Akt signaling pathway and Wnt signaling pathway, fat-metabolism-related pathways such as primary bile acid biosynthesis and pancreatic secretion. Eleven pathways directly related to fat metabolism were also obtained (Figure 4A,B). These genes were involved in primary bile acid biosynthesis, fatty acid elongation, biosynthesis of unsaturated fatty acid, and the metabolism of various types of lipids. DEGs in the B vs. D group were significantly enriched (*p* < 0.05) in 11 signaling pathways, including the calcium signaling pathway, Wnt signaling pathway, PPAR signaling pathway, bile and pancreatic secretion, and the regulation of lipolysis in adipocytes. In addition, six pathways directly related to fat metabolism were also selected (Figure 4C,D). These genes were involved in the regulation of lipolysis in adipocytes, primary bile acid biosynthesis, biosynthesis of unsaturated fatty acids, and lipid metabolism, etc. Fifteen genes, including SCD, PNPLA3, FGF7, ADCY2, WNT11 and ACOT8, were selected as candidate genes to regulate fat deposition (Table 3).

### 3.6. Validation of DEGs

**Figure 5:**
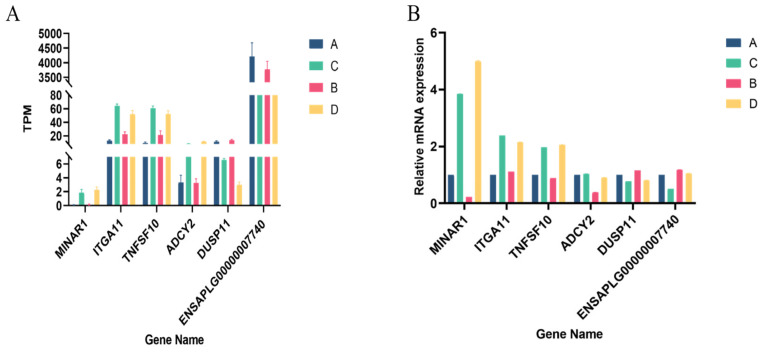
qRT-PCR validation of DEGs: (**A**) TPM of six randomly selected genes; and (**B**) the relative expression of six randomly selected genes.

Six randomly selected differentially expressed genes were subjected to qRT-PCR to verify the RNA sequencing results. As shown in Figure 5, all the selected DEGs showed concordant expression patterns between the RNA-Seq and qRT-PCR results, indicating the true reliability of our sequencing and analysis methods.

## 4. Discussion

In this study, although there was no significant difference in body weight between the two strains of Pekin ducks, the fat ratio of Jingdian ducks was significantly lower than that of Nankou 1, which fully demonstrated that the fat deposition capacity of Nankou 1 ducks was stronger than that of Jingdian ducks. Subcutaneous fat deposition is an important economic trait in meat ducks. Interestingly, according to relevant reports, the sebum rates of Nankou 1 ducks averaged around 30% [18], while the sebum rates of Jingdian ducks remained above 33%, contrary to the results of this study. We speculate that on the one hand, the current commercial feeding of Nankou 1 still adopts force-feeding, while in this experiment free feeding of high energy feed was adopted, which avoided the stress response and body damage caused by force-feeding and contributed to duck development and fat accumulation. On the other hand, Nankou 1 has been on the market for more than ten years after successful breeding, and its subcutaneous fat deposition ability has been greatly improved after continuous screening. 

We performed transcriptome analysis of subcutaneous adipose tissue to screen for candidate genes regulating lipid deposition. These genes enhanced fat deposition capacity by promoting fat synthesis or inhibiting lipolysis. SCD (Stearoyl-CoA desaturase) is the rate-limiting enzyme that catalyzes the conversion of saturated fatty acids (SFA) into monounsaturated fatty acids (MUFA), and its synthesized MUFAs are the main substrates for the synthesis of various types of lipids, including phospholipids, triglycerides, and cholesteryl esters [19]. Lower levels of MUFA can limit fat accumulation [20]. Mice with targeted deletion of the SCD gene exhibited reduced body fat rate, increased insulin sensitivity, and resistance to obesity induced by a high-fat or high-carbohydrate diet [21,22]. Recent studies have shown that the inhibition of SCD promoted the fatty acid tendency to oxidative pathways in vivo [23], and that the regulation of SCD expression through the peroxisome proliferator-activated receptor (PPAR) pathway could regulate the synthesis of intramuscular fat and abdominal fat in broilers [24]. Therefore, we hypothesized that the upregulation of SCD expression in duck subcutaneous adipose tissue can not only promote fatty acid synthesis, but also inhibit fatty acid oxidation, thereby achieving the effect of increased fat deposition. FGF7 (Fibroblast growth factor 7) is a member of the fibroblast growth factor family, and most members of this family can facilitate the proliferation and differentiation of human preadipocytes by activating the receptor tyrosine kinase family [25]. A study on the development of adipocytes in chickens showed that adipogenesis could be promoted by inhibiting FGF7 expression in adipose tissue, which may be caused by biased differentiation and the reduced proliferation of preadipocytes [25,26]. Some scholars have analyzed the abdominal fat of lean and fat broilers through genome-wide association studies (GWAS) and found that FGF7 was significantly correlated with abdominal fat weight and was a key gene regulating abdominal fat deposition [27]. The results of this study showed that FGF7 expression was downregulated in duck adipose tissue with strong fat deposition capacity. We speculated that the inhibition of FGF7 expression promoted the differentiation of preadipocytes and improved the efficiency of fat synthesis, but the specific mechanism still needs further study. Transforming growth factor-beta (TGFβ) is a class of cytokines that is activated by binding to propeptides and latent TGFβ binding protein (LTBP) and has multiple effects on cell development. TGFβ has been shown to inhibit adipocyte differentiation and thereby lipogenesis [28]. A study on the regulation of lipogenesis by LTBP and TGFβ showed that elevated levels of active TGFβ were detected in LTBP-deleted cells [29]. This result once again demonstrated that LTBP regulated TGFβ activation and then promoted adipocyte differentiation by inhibiting the TGFβ signaling pathway. In the present study, ducks with high sebum rates showed a significant downregulation of LTBP expression, which means that the reduced LTBP expression decreased the active TGFβ content, attenuated the inhibitory effect of TGFβ on adipocyte differentiation, and promoted fat synthesis.

Approximately 90% of the adipocyte volume is occupied by a lipid droplet (LD) containing triglyceride (TAG) [30], and the lipid-droplet-associated protein PLIN1 protects TAG from breakdown by lipase [31]. TAG is hydrolyzed when body energy requirements increase [32]. Adipose triglyceride lipase (ATGL), hormone-sensitive lipase (HSL), and monoglycerol lipase (MGL) [33] catalyze the hydrolysis of TAG, diacylglycerol (DAG), and monoacylglycerol (MAG), respectively [34]. The activity of ATGL is regulated by an activating protein, comparative gene identification 58 (CGI-58) [34]. Patatin-like phospholipase domain-containing 3 (PNPLA3), a LD-related protein, can directly bind to CGI-58 [35], which can achieve the effect of inhibiting ATGL-catalyzed TAG decomposition [36,37]. Adenylate cyclase encoded by ADCY2 (Adenylate cyclase 2) can catalyze the conversion of ATP to the second messenger cyclic adenosine monophosphate (cAMP) [38]. The cAMP-dependent protein kinase A (PKA) pathway is the main pathway of lipolysis, ADCY catalyzes intracellular cAMP production, which activates PKA, and activates PKA phosphorylates HSL and PLIN1 in the cytoplasm [39], so that HSL can enter the LD to participate in lipolysis [40]. At the same time, phosphorylation of PLIN1 can promote the release of CGI-58, which is conducive to the activation of ATGL [41]. This suggests that the activation of ADCY2 promotes lipolysis within adipocytes via the cAMP-PKA signaling pathway. According to reports, GWAS analysis of the subcutaneous fat of obese people revealed that the best model affecting genetic variation in subcutaneous fat included 7 genes, including ADCY2 [42], which further demonstrated that ADCY2 could regulate subcutaneous fat deposition. In this study, the transcriptome results showed that the expression of PNPLA3 was significantly upregulated, while the expression of ADCY2 was significantly downregulated in the subcutaneous fat of Nankou 1 Pekin ducks. Therefore, we speculated that increasing PNPLA3 expression and decreasing ADCY2 expression could reduce ATGL and HSL enzyme activities, inhibit TAG and DAG hydrolysis, and then achieve the effect of increasing subcutaneous fat deposition. Acyl-CoA thioesterase (ACOT) is a group of enzymes that catalyze the hydrolysis of fatty acyl-CoA to free fatty acids (FFAs) and coenzyme A (CoASH) and has the potential to regulate the intracellular levels of fatty acyl-CoA, FFAs, and CoASH [43]. ACOT8 is highly conserved and hydrolyzes mainly medium-chain to long-chain fatty acyl-CoA [44]. The expression of ACOT8 could be regulated by PPARα, and since PPARα was involved in regulating mitochondrial β oxidation, it was speculated that ACOT8 had a regulatory role in fatty acid oxidation [45]. Existing studies have confirmed that ACOT8 catalyzes the hydrolysis of all fatty acyl-CoA [46], and that its activity is regulated by PPARα and CoASH levels [43,44]. Based on the available research results and the results of this trial, we hypothesized that upregulation of ACOT8 suppressed fatty acid synthesis and enhanced β oxidation, thereby inhibiting fat synthesis [47].

In addition to the above candidate genes affecting fat deposition by regulating fat synthesis and lipolysis, we also found that WNT11, CH25H, ITGA11, THSD4, LPAR1 and LGR5 might be involved in the regulation of subcutaneous fat deposition in ducks. However, the links and specific mechanisms involved in fat metabolism still need to be further studied.

## 5. Conclusions

Biological statistics showed that there were significant differences in fat deposition between Nankou 1 and Jingdian Pekin ducks. By identifying DEGs and conducting enrichment analysis, six key candidate genes, including SCD, FGF7, LTBP1, PNPLA3, ADCY2, and ACOT8, were selected. The results of this study provide a foundation for exploring the molecular mechanism of subcutaneous fat deposition regulation in ducks.

## Figures and Tables

**Table 1:** Genes used for qRT-PCR and their primers.

Gene	Primer Sequences (5′ to 3′)
ADCY2	F: GCGAGCGGCGAGCAGTC
	R: TGAGGAGCAGGAAGACGAGGAG
ITGA11	F: ACAACCGCAACCTCACCATCC
	R: ACACCATCACCGTTCACATCCAG
MINAR1	F: GAGGCAGACAGGCAATACGAAATC
	R: GCAGGGTAGGGATGAGGACTAAAG
TNFSF10	F: GCCGTCACCTTCCTCTACTTCAC
	R: AAATCTCCAAGTTCCTCCCCAGTG
DUSP11	F: AGAATTTGGGCTTGGACCTCCTC
	R: CTTGCTTGCGGTTCTTCTTGGTAG
ENSAPLG00000007740	F: GATGCGGGCGTGGGAGTG
	R: GATGAGGAACTGTGGAAGCAAAGC
GAPDH	F: AGTGAAGGCTGCTGCTGATGG
	R: TCAAAGGTGGAGGAATGGCTGTC

**Table 2:** Effects of strain and sex on slaughter performance of Pekin ducks ^1^.

Items	Nankou 1		Jingdian		*p*-Value from ANOVA		
	Female	Male	Female	Male	Strain	Sex	Strain × Sex
Live weight (kg)	3.94 ± 0.05	4.01 ± 0.05	3.84 ± 0.03	4.00 ± 0.05	0.252	0.020	0.360
Eviscerated yield (%)	74.68 ± 1.32	74.59 ± 1.26	74.17 ± 1.10	76.83 ± 0.68	0.447	0.259	0.228
Breast muscle yield (%)	12.17 ± 0.39 ^A^	11.75 ± 0.28 ^Aa^	14.17 ± 0.36 ^B^	13.06 ± 0.24 ^b^	<0.001	0.024	0.296
Thigh muscle yield (%)	9.35 ± 0.36	9.67 ± 0.14	9.58 ± 0.16	9.46 ± 0.34	0.976	0.710	0.412
Abdominal fat yield (%)	3.28 ± 0.14 ^A^	3.06 ± 0.13	3.25 ± 0.10 ^a^	2.60 ± 0.18 ^Bb^	0.100	0.005	0.141
Subcutaneous fat yield (%)	39.52 ± 1.14	40.61 ± 2.26 ^a^	38.42 ± 1.12	34.02 ± 1.29 ^b^	0.018	0.287	0.083

^1^ Data represent 4 replicates, 2 ducks per replicate. Different lowercase letters indicate significant differences (*p* < 0.05), different superscript capital letters indicate extremely significant differences (*p* < 0.01).

**Table 3:** Candidate genes that regulate fat deposition.

Gene_id	Gene Name	Gene Description	Log2FC(A/C)	*p*-Adjust	Log2FC(B/D)	*p*-Adjust
ENSAPLG00000002172	CH25H	Cholesterol 25-hydroxylase	1.694446363	0.002958384	——	——
ENSAPLG00000003497	——	——	−1.78896802	2.31 × 10^−23^	1.451332565	2.70 × 10^−12^
ENSAPLG00000003663	ITGA11	Integrin subunit alpha 11	−1.74401932	3.14 × 10^−32^	1.368413056	3.71 × 10^−7^
ENSAPLG00000004328	FGF7	Fibroblast growth factor 7	1.169631738	3.31 × 10^−8^	1.388239511	0.000132701
ENSAPLG00000008644	ADCY2	Adenylate cyclase 2	1.136973915	0.023019894	1.471927986	0.004665066
ENSAPLG00000010035	LPAR1	lysophosphatidic acid receptor 1	1.559385156	3.03 × 10^−7^	1.238075251	0.011128412
ENSAPLG00000010423	THSD4	Thrombospondin type 1 domain containing 4	1.570837435	7.39 × 10^−6^	−1.41148092	9.37 × 10^−8^
ENSAPLG00000010434	——	——	5.565023239	0.009097501	−3.39527429	0.004851312
ENSAPLG00000010439	WNT11	Wnt family member 11	1.208117664	0.000584256	1.513587588	6.41 × 10^−5^
ENSAPLG00000012621	LTBP1	Latent transforming growth factor beta binding protein 1	1.815485766	1.54 × 10^−22^	1.431284807	0.000190098
ENSAPLG00000015737	LGR5	Leucine rich repeat containing G protein-coupled receptor 5	2.443874812	1.48 × 10^−6^	1.910981744	1.99 × 10^−6^
ENSAPLG00000023048	ACOT8	Acyl-CoA thioesterase 8	5.889463356	0.004179155	7.347192617	0.003168815
ENSAPLG00000030592	——	——	2.434530022	0.047749977	7.022715461	1.32 × 10^−68^
ENSAPLG00000015665	SCD	Stearoyl-CoA desaturase	——	——	2.646755213	5.92 × 10^−15^
ENSAPLG00000002630	PNPLA3	Patatin like phospholipase domain containing 3	——	——	1.651888747	1.38 × 10^−15^

## Data Availability

The original contributions presented in the study are publicly available. All sequencing data are available through the NCBI Sequence Read Archive under the accession number PRJNA1034243.

## Supplementary Materials

The following supporting information can be downloaded at: https://www.mdpi.com/article/10.3390/ani14020268/s1, Table S1: Statistics of sequencing data; Table S2: Comparison results between cleaned data and reference genome; Table S3: DEGs between group A and C; Table S4: DEGs between group B and D; Table S5: DEGs associated with fat metabolism from A vs. C group; Table S6: DEGs associated with fat metabolism from B vs. D group.
